# Differential privacy in the 2020 US census: what will it do? Quantifying the accuracy/privacy tradeoff

**DOI:** 10.12688/gatesopenres.13089.2

**Published:** 2020-04-06

**Authors:** Samantha Petti, Abraham Flaxman

**Affiliations:** 1 School of Mathematics, Georgia Institute of Technology, Atlanta, GA, 30332, USA; 2 Institute for Health Metrics and Evaulation, University of Washington, Seattle, Seattle, WA, 98121, USA

**Keywords:** Decennial census, differential privacy, TopDown algorithm, empirical privacy loss

## Abstract

**Background:** The 2020 US Census will use a novel approach to disclosure avoidance to protect respondents’ data, called TopDown. This TopDown algorithm was applied to the 2018 end-to-end (E2E) test of the decennial census. The computer code used for this test as well as accompanying exposition has recently been released publicly by the Census Bureau.

**Methods:** We used the available code and data to better understand the error introduced by the E2E disclosure avoidance system when Census Bureau applied it to 1940 census data and we developed an empirical measure of privacy loss to compare the error and privacy of the new approach to that of a (non-differentially private) simple-random-sampling approach to protecting privacy.

**Results:** We found that the empirical privacy loss of TopDown is substantially smaller than the theoretical guarantee for all privacy loss budgets we examined. When run on the 1940 census data, TopDown with a privacy budget of 1.0 was similar in error and privacy loss to that of a simple random sample of 50% of the US population. When run with a privacy budget of 4.0, it was similar in error and privacy loss of a 90% sample.

**Conclusions:** This work fits into the beginning of a discussion on how to best balance privacy and accuracy in decennial census data collection, and there is a need for continued discussion.

## Acronyms

DP - differentially private

E2E - end-to-end

TC - total count

SC - stratified count

MAE - median absolute error

EPL - empirical privacy loss

## Introduction

In the United States, the Decennial Census is an important part of democratic governance. Every ten years, the US Census Bureau is constitutionally required to count the “whole number of persons in each State,” and in 2020 this effort is likely to cost over 15 billion dollars ^1, 2^. The results will be used for apportioning representation in the US House of Representatives and dividing federal tax dollars between states, as well as for a multitude of other governmental activities at the national, state, and local levels. Data from the decennial census will also be used extensively by sociologists, economists, demographers, and other researchers, and it will also inform strategic decisions in the private and non-profit sectors, and facilitate the accurate weighting of subsequent population surveys for the next decade ^3^.

The confidentiality of information in the decennial census is also required by law, and the 2020 US Census will use a novel approach to “disclosure avoidance” to protect respondents’ data ^4^. This approach builds on Differential Privacy, a mathematical definition of privacy that has been developed over the last decade and a half in the theoretical computer science and cryptography communities ^5^. Although the new approach allows a more precise accounting of the variation introduced by the process, it also risks reducing the utility of census data—it may produce counts that are substantially less accurate than the previous disclosure avoidance system, which was based on redacting the values of table cells below a certain size (cell suppression) and a technique called swapping, where pairs of households with similar structures but different locations had their location information exchanged in a way that required that the details of the swapping procedure be kept secret ^6^.

To date, there is a lack of empirical examination of the new disclosure avoidance system, but the approach was applied to the 2018 end-to-end (E2E) test of the decennial census, and computer code used for this test as well as accompanying exposition has recently been released publicly by the Census Bureau ^4, 7^.

We used the recently released code, preprints, and data files to understand and quantify the error introduced by the E2E disclosure avoidance system when the Census Bureau applied it to 1940 census data (for which the individual-level data has previously been released ^8^) for a range of privacy loss budgets. We also developed an empirical measure of privacy loss and used it to compare the error and privacy of the new approach to that of a (non-differentially private) simple-random-sampling approach to protecting privacy.

## Methods

### Differential privacy definition and history

A randomized algorithm for analyzing a database is differentially private (DP) if withholding or changing one person’s data does not substantially change the algorithm’s output. If the results of the computation are roughly the same whether or not my data are included in the database, then the computation must be protecting my privacy. DP algorithms come with a parameter *ϵ*, which quantifies how much privacy loss is allowed, meaning how much can one person’s data to affect the analysis.

To be precise, a randomized algorithm is *ϵ*-DP if, for each possible output , for any pair of datasets *D * and *D'* that are the same everywhere except for on one person’s data,

$$\text{Pr}\left[A\left(D\right)=P\right]\leq \text{exp}\left(\epsilon \right)\text{Pr}\left[A\left(D^{\prime }\right)=P\right].$$

Differential privacy is a characteristic of an algorithm; it is not a specific algorithm. Algorithms often achieve differential privacy by adding random variation ^5^.

The new disclosure avoidance system for the 2020 US Census is designed to be DP and to maintain the accuracy of census counts. To complicate things beyond the typical challenge faced in DP algorithm design, there are certain counts in the census that will be published precisely as enumerated, without any variation added. These invariants have not been selected for the 2020 decennial census yet, but in the 2018 end-to-end (E2E) test, the total count for each state and the number of households in each enumeration district were invariants. There are also inequalities that will be enforced. The E2E test required the total count of people in an enumeration district to be greater or equal to the number of occupied households in that district ^9^.

### TopDown algorithm

At a high level, the census approach to this challenge repeats two steps for multiple levels of a geographic hierarchy (from the top down, hence their name “TopDown”). The first step (Imprecise Histogram) adds variation from a carefully chosen distribution to the stratified counts of individuals. This produces a set of counts with illogical inconsistencies, which we refer to as an “imprecise histogram”. For example, counts in the imprecise histogram might be negative, might violate invariants or other inequalities, or might be inconsistent with the counts that are one level up in the geographic hierarchy. The second step (Optimize) finds optimized counts for each most-detailed cell in the histogram, using constrained convex optimization to make them as close as possible to the counts in the imprecise histogram, subject to the constraints that the optimized counts be non-negative, consistent with each other and the higher levels of the hierarchy, and satisfy the invariants and inequalities. These two steps are performed for each geographic level, from the coarsest to the finest. Each level is assigned a privacy budget *ϵ*_i_ (which governs how much variation to add in the Imprecise Histogram step), and the entire algorithm achieves *ϵ*-DP for $\epsilon =\sum_{i}^{}\epsilon_{i}.$ The 2020 US Census data may have six geographic levels, nested hierarchically: national, state, county, census tracts, block groups, and blocks; but in the 1940 E2E test four levels (national, state, county, and enumeration district) were included.

***Step one: Imprecise Histogram.*** In the E2E algorithm applied to the 1940s microdata, TopDown added random variation in a flexible way that allowed the user to choose what statistics are the most important to keep accurate. The variation was added to the detailed histogram counts for the level and also to a preselected set of aggregate statistics. The detailed histogram counts stratified the population of each geographic by age (two values: under-18-year-olds and 18-plus), race (six values), ethnicity (two values: Hispanic and non-Hispanic), and household/group-quarters type (6 values). The aggregate statistics are sets of histogram count sums specified by some characteristics. For example, the “race/ethnicity/age” aggregate statistic contains 24 counts: people of each of the six racial categories who are also Hispanic ethnicity under age 18, of Hispanic ethnicity age 18 and over, of non-Hispanic ethnicity under age 18, and of non-Hispanic ethnicity age 18 and over.

The aggregate statistics (internally called “DP queries” in the TopDown algorithm) afford a way to choose specific statistics that are more important to keep accurate, and the E2E test included two such aggregates: a household/group-quarters query, which increases the accuracy of the count of each household type at each level of the hierarchy, and a race/ethnicity/age query, which increases the accuracy of the stratified counts of people by race, ethnicity, and voting age across all household/group-quarters types (again for each level of the spatial hierarchy). It also included “detailed queries” corresponding to boxes in the histogram. The detailed queries were afforded 10% of the privacy budget at each level, while the DP queries split the remaining 90% of the privacy budget, with 22.5% spent on the household/group-quarters queries and 67.5% spend on the race/ethnicity/age queries.

The epsilon budget of the level governed how much total random variation to add. A further parameterization of the epsilon budget determined how the variance was allocated between the histogram counts and each type of aggregate statistic. We write *ϵ _i_* = *h* + *s*_1_+ *s*_2_ +...+ *s _k_*, where *ϵ*_i_ was the budget for the geographic level, *h* was the budget for the detailed queries, and *s*_1_,... *s _k_* were the budgets for each of the *k* types of aggregate statistics. Then variance was added independently to each count according to the follow distribution:

imprecisedetailedhistogramcount=precisedetailedhistogramcount+G(h/2)

$$\text{imprecise}\;\text{aggregate}\;\text{stat}\;j=\text{precise}\;\text{aggregate}\;\text{stat}\;j+G\left(s_{j}/2\right)$$

where *G(z)* denotes the two-tailed geometric distribution,

$$\text{Pr}\left[G\left(z\right)=k\right]=\frac{\left(1-\text{exp}\left(-z\right)\right)\text{exp}\left(-zk\right)}{1+\text{exp}\left(-z\right)}.$$

The imprecise counts and imprecise aggregate statistics are unbiased estimates with variance (1 – exp(– *z*)) ^2^/(2exp(– *z*)), where *z* is the parameter for the geometric random variable added. A higher privacy budget means the variance added is more concentrated around zero, and therefore the corresponding statistic is more accurate. Therefore, adjusting the privacy budgets of the various aggregate statistics gives control over which statistics are the most private/least accurate (low fraction of the budget) and the most accurate/least private (high fraction of the budget).

The variation added to each histogram count comes from the same distribution, and is independent of all other added variation; the variance does not scale with the magnitude of count, e.g. adding 23 people to the count of age 18 and older non-Hispanic Whites is just as likely as adding 23 people to the count of age under 18 Hispanic Native Americans, even though the population of the latter is smaller.

***Step two: Optimize.*** In this step, the synthetic data is created from the imprecise detailed histogram counts and aggregate statistics by optimizing a quadratic objective function subject to a system of linear equations and inequalities. The algorithm creates a variable for each detailed histogram count and each aggregate statistic. It adds equations and inequalities to encode the requirements that (i) each count and aggregate statistic is non-negative, (ii) the invariants and inequalities are satisfied, (iii) the aggregate statistics are the sum of the corresponding detailed histogram counts, and (iv) the statistics are consistent with the higher level synthetic data counts (i.e. the total number of people aged 18 and over summed across the counties in a state is equal to the number of people aged 18 and over in that state as reported by synthetic data set constructed in the previous phase). The optimization step finds a solution that satisfies these equations and minimizes the weighted sum of the squared differences between each variable/aggregate of variables and the corresponding imprecise detailed histogram count or imprecise aggregate statistic. This sum is weighted with the weight of each term taken to be proportional to the magnitude of the variation added in step one to create the imprecise count. The solution to this optimization is not necessarily integral, however, and TopDown uses a second optimization step to round fractional counts to integers.

We note that the approach that Census Bureau has taken with the TopDown where imprecise histogram data is optimized based on internal consistency has been developed in a line of research over the last decade to that has focused on obtaining count data that is DP *and* accurate ^10– 13^.

### Empirical Privacy Loss for quantifying impact of optimize steps

As described above, the privacy loss of a DP algorithm is quantified by a unitless number, *ϵ*, that bounds the maximum of the log of the relative change in the probability of an output when one person’s data is changed. This bound is typically proven by logical deduction, and for complex DP algorithms, the proof often relies on the Sequential Composition Theorem ^5^, which states that information derived by combining the output of an *ϵ*_1_-DP algorithm and an *ϵ*_2_-DP algorithm is at most ( *ϵ*_1_ + *ϵ*_2_)-DP. This theorem is an inequality, however, and the inequality might have room for improvement.

It is possible to empirically quantify privacy loss, which has the potential to show that the inequality of the sequential composition theorem is not tight. The brute force approach quantify privacy loss empirically is to search over databases *D* and *D'* that differ on one row to find the event *E* with the largest ratio of probabilities; this is too computationally intensive to be feasible for all but the simplest DP algorithms.

For algorithms that produce DP counts of multiple subpopulations, such as TopDown, it is possible to use the distribution of the residual difference between the precise count and the DP count to derive a proxy of the distribution produced by the brute force approach ^14^. The special structure of count queries affords a way to avoid re-running the algorithm repeatedly, which is essential for TopDown, since it takes several hours to complete a single run of the algorithm. Assuming that the residual difference of the DP count minus the precise count is identically distributed for queries across similar areas (such as voting-age population across all enumeration districts), and then instead of focusing on only the histogram counts containing the individual who has changed, we used the residuals for all areal units to estimate the probability of the event we are after:

$$\text{Pr}\left[{\text{error}}_{j}=k\right]\approx \left(\sum_{j^{\prime }=1}^{c}1\left[\left\{{\text{error}}_{j^{\prime }}=k\right\}\right]\right)/C=:{\hat{p}}_{k},$$

where error _*j*_ is the residual difference of DP counts returned by TopDown minus the precise count for that same quantity in the 1940 census, and the error _*j'*_ are residuals for *C* other queries assumed to be exchangeable.

To measure the empirical privacy loss (EPL), we approximated the probability distribution of the residuals (DP count minus precise count at a selected level of the geographic hierarchy), which we denote *p* ^KDE^( *x*) , using Gaussian kernel density estimation (KDE) with a bandwidth of 0.1, and compare the log-ratio inspired by the definition of *ϵ*-DP algorithms:

$$\text{EPL}\left(x\right)=\text{log}\left(\frac{p^{\text{KDE}}\left(x\right)}{p^{\text{KDE}}\left(x+1\right)}\right);$$

$$\text{EPL}=\underset{x\in \left(-\infty ,\infty \right)}{\text{max}}\left\{\text{abs}\left(\text{EPL}\left(x\right)\right)\right\}$$

See Supplementary Methods Appendix for additional detail on the design and validation of the EPL metric ^15^.

## TopDown options still to be selected

There are seven key choices in implementing TopDown, that balance accuracy and privacy. We list them here, and state how they were set in the 2018 end-to-end test when run on the 1940s Census data:

1. Overall privacy. A range of *ϵ* values, with {0.25, 0.50, 0.75, 1.0, 2.0, 4.0, 8.0} used in the E2E test run on the 1940 Census Data.
2. How to split this budget between national, state, county, tract, block group, and block. In the test run, *ϵ* was split evenly between national, state, county, and enumeration district.
3. What aggregate statistics (also known as “DP Queries”) to include. In the test, two DP Queries were included: (i) counts stratified by age-group/race/ethnicity (and therefore aggregated over household/group-quarters type); and (ii) the household/group-quarters counts, which tally the total number of people living in each type of housing (in a household, in institutional facilities of certain types, in non-institutional facilities of certain types).
4. At each level, how to split level-budget between detailed queries and DP queries. The test run used 10% for detailed queries, 22.5% for household/group-quarters; and 67.5% for age-group-/race-/ethnicity-stratified counts.
5. What invariants to include. The test run held the total population count at the national and state level invariant.
6. What constraints to include. The test run constrained the total count of people to be greater or equal to total count of occupied households at each geographic level.
7. What to publish. The test run published a synthetic person file and synthetic household file for a range of *ϵ* values, for four different seeds to the pseudorandom number generator.

### Our evaluation approach

1. We calculated residuals (DP count minus precise count) and summarized their distribution by its median absolute error (MAE) for total count (TC) and age/race/ethnicity stratified count (SC) at the state, county, and enumeration-district level. We also summarized the size of these counts from the precise-count versions to understand relative error as well as the absolute error introduced by TopDown.
2. We calculated a measure of empirical privacy loss (EPL), inspired by the definition of differential privacy. To measure EPL, we approximated the probability distribution of the residuals (DP count minus precise count at a selected level of the geographic hierarchy), which we denote *p* ^KDE^( *x*), using Gaussian kernel density estimation with a bandwidth of 0.1, and compare the log-ratio inspired by the definition of *ϵ*-DP algorithms:
  $$\text{EPL}\left(x\right)=\text{log}\left(\frac{p^{\text{KDE}}\left(x\right)}{p^{\text{KDE}}\left(x+1\right)}\right);$$
  $$\text{EPL}=\underset{x\in \left(-\infty ,\infty \right)}{\text{max}}\left\{\text{abs}\left(\text{EPL}\left(x\right)\right)\right\}$$
  See Supplementary Methods Appendix for additional detail on the design and validation of the EPL metric ^15^. We hypothesized that the EPL of TopDown will be substantially smaller than the theoretical guarantee of *ϵ*, which was proven using the Sequential Composition Theorem, which provides an inequality that is usually not a tight bound ^14^. However, it is possible that it will be much larger than *ϵ*, due to the difficult-to-predict impact of including certain invariants.
3. We searched for bias in the residuals from (1), with our hypothesis that the DP counts are larger than precise counts in spatial areas with high homogeneity and DP counts are smaller than precise counts in areas with low homogeneity. We based this hypothesis on the expected impact of the non-negativity constraints included in the optimization steps of the TopDown algorithm. For each detailed query with a negative value for its noisy count, the optimization step will increase the value to make the results logical, and this reduction in variance must tradeoff some increase in bias. To quantify the scale of the bias introduced by optimization, for each geographic area, we constructed simple homogeneity index by counting the cells of the detailed histogram that contained a precise count of zero, and we examined the bias, defined as the mean of the DP count minus precise count, for these areas when stratified by homogeneity index.
4. We also compared the median absolute error and empirical privacy loss of TopDown to a simpler, but not-differentially-private approach to protecting privacy, Simple Random Sampling (i.e. sampling without replacement) for a range of sized samples. To do this, we generated samples without replacement of the 1940 Census Data for a range of sizes, and applied the same calculations from (1) and (2) to this alternatively perturbed data.

## Results

### Error and privacy of TopDown

Recall that geographic areas are nested: enumeration districts are contained within counties, which are contained within states. We found error in total count (TC) varied as a function of total privacy loss budget. Running TopDown with *ϵ* = 0.5 produced median absolute error in TC of 29 at the enumeration district level and 45 at the county level; *ϵ* = 1.0 produced median absolute error in TC of 15 at the enumeration district level and 24 at the county level; and *ϵ* = 2.0 produced median absolute error in TC of 8 at the enumeration district level and 13 at the county level (Full table in Extended Data ^16^). At the state level, there was TC error of 0.0, as expected from the state TC invariant. The median and 95th percentile of TC from the precise-count data were 865 and 2342 for enumeration districts, 18,679 and 122,710 for counties, and 1,903,133 and 7,419,040 for states.

**Figure 1.**
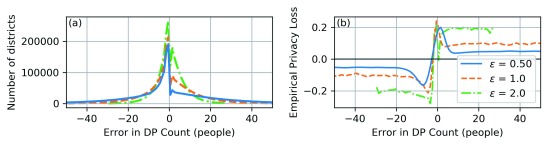
Error distribution and empirical privacy loss for stratified counts at the enumeration district level. Panel (a) shows the distribution of residuals (DP - Precise) for stratified counts at the enumeration district level, stratified by age, race, and ethnicity; and panel (b) shows the empirical privacy loss function, $\text{EPL}(x)=\text{log}({\hat{p}}^{\text{KDE}}(x)/{\hat{p}}^{\text{KDE}}(x+1)),\;$ where $\hat{p}(x)$ is the probability density corresponding to the histogram in (a), after smoothing with a Gaussian kernel of bandwidth 0.1; the EPL value is the maximum of the absolute value of EPL( *x*) over all *x*.

Error in stratified count (SC) varied similarly; when *ϵ* = 0.5, the median absolute error in SC at the enumeration district level was 10 people, at the county level was 11 people, and at the state level was 13 people; for *ϵ* = 1.0, the median absolute error in SC at the enumeration district level was 6 people, at the county level was 6 people, and at the state level was 7 people; and for *ϵ* = 2.0, the median absolute error in SC at the enumeration district level was 4 people, at the county level was 4 people, and at the state level was 4 people. The median and 95th percentile of SC from the precise-count data were 88 and 967 for enumeration districts, 47 and 17,480 for counties, and 229 and 714,208 for states. ( Figure 1)

We found that the empirical privacy loss was often substantially smaller than the privacy loss budget. For *ϵ* = 0.5, the empirical privacy loss for TC at the enumeration district level was 0.033 and at the county level was 0.035 (at the state level empirical privacy loss is undefined, since the invariant makes all residuals zero); for *ϵ* = 1.0, the empirical privacy loss for TC at the enumeration district level was 0.064 and at the county level was 0.048; and for *ϵ* = 2.0, the empirical privacy loss for TC at the enumeration district level was 0.116 and at the county level was 0.094.

This relationship between privacy loss budget and empirical privacy loss was similar for stratified counts (SC) at the enumeration district and county level, but for privacy loss budgets of 1.0 and less, the empirical privacy at the enumeration district level was loss for SC was not as responsive to *ϵ*. For *ϵ* = 1.5, the empirical privacy loss for SC at the enumeration district level was 0.200, at the county level was 0.165, and at the state level was 0.104; for *ϵ* = 1.0, the empirical privacy loss for SC at the enumeration district level was 0.241, at the county level was 0.164, and at the state level was 0.166; and for *ϵ* = 2.0, the empirical privacy loss for SC at the enumeration district level was 0.280, at the county level was 0.253, and at the state level was 0.300. EPL values for all combinations of *ϵ* and all geographic levels appear in the Extended Data.

### Comparison with error and privacy of simple random sampling

We found that the MAE and EPL of Simple Random Sampling (i.e. sampling uniformly, without replacement) varied with larger sample size in a manner analogous to the total privacy budget in TopDown, for *ϵ* ≥ 1. For a 5% sample of the 1940 Census data, we found median absolute error in TC of 74 at the enumeration district level, 388 at the county level, and 3883 at the state level; a 50% sample produced median absolute error in TC of 17 at the enumeration district level, 90 at the county level, and 932 at the state level; and a 95% sample produced median absolute error in TC of 4 at the enumeration district level, 20 at the county level, and 130 at the state level.

Error in stratified count varied similarly; for a 5% sample, we found median absolute error in SC of 18 at the enumeration district level, 19 at the county level, and 41 at the state level; a 50% sample produced median absolute error in TC of 4 at the enumeration district level, 5 at the county level, and 9 at the state level.

**Figure 2.**
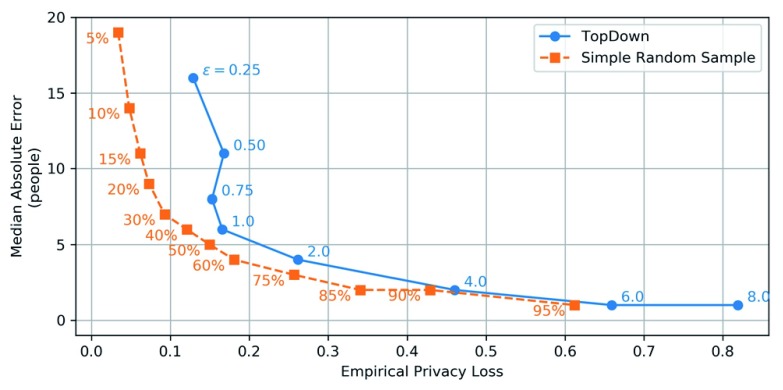
Tradeoff curve of median absolute error and empirical privacy loss of stratified counts at the county level. The curve with circular markers shows that in TopDown, the choice of *ϵ* controls the tradeoff between MAE and EPL, although for *ϵ* < 1 there is not much difference in EPL. The curve with square markers shows the MAE and EPL of Simple Random Sampling for a range of sample sizes, for comparison. For example, TopDown with *ϵ* = 1.0. provides privacy loss and estimation error similar to a sample of 50% of the 1940 census data, while *ϵ* = 2.0 is comparable to a 75% sample (for counts stratified by age, race, and ethnicity at the county level; different aggregate statistics produce different comparisons).

We found empirical privacy loss increased as sample size increased. For a 5% sample, at the enumeration district level, we found EPL of 0.020 for TC and 0.098 for SC, and at the county level, we found 0.035 for TC and 0.034 for SC; a 50% sample produced EPL of 0.079 for TC and 0.318 for SC at the enumeration district level, and 0.082 for TC and 0.150 for SC at the county level; and a 95% sample produced EPL of 0.314 for TC and 1.333 for SC at the enumeration district level, and 0.429 for TC and 0.612 for SC at the county level ( Figure 2, Table 1).

**Table 1.** Values of privacy loss, and corresponding proportions of Simple Random Sample (SRS) with most similar median-absolute-error/empirical-privacy-loss profile.

Privacy Budget ( *ϵ*)	Closest SRS sample proportion (%)
1.0	50%
2.0	75%
4.0	90%
6.0	95%

### Bias in the variation introduced by TopDown

**Figure 3.**
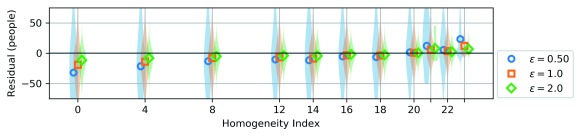
Relationship between homogeneity index and residual for three values of epsilon. The homogeneity index, defined as the number of cells with precise count of zero in the detailed histogram, is positively associated with the bias (markers show the mean difference between the DP count estimated by TopDown and the precise count, and shaded area shows the distribution of individual differences). This plot shows the association for enumeration districts, and a similar relationship holds at the county level. As *ϵ* increases, the scale of the bias decreases. (Enumeration districts attained only a subset of the homogeneity index values between 0 and 23, which is why there are different width gaps between markers. We pooled the residuals for the four runs of TopDown with different random seed.)

The bias introduced by TopDown varied with homogeneity index, as hypothesized. Enumeration districts with homogeneity index 0 (0 empty cells in the detailed histogram) had TC systematically lower than the precise count, while enumeration districts homogeneity index 22 (the maximum number of empty cells observed in the detailed histogram) had TC systematically higher than the precise count. The size of this bias decreased as a function of *ϵ*. Homogeneity index 0 had bias of -31.7 people for *ϵ* = 0.5, -18.9 people for *ϵ* = 1.0, and -11.6 people for *ϵ* = 2.0; while homogeneity index 22 had bias of 5.4 people for *ϵ* = 0.5, 3.6 people for *ϵ* = 1.0, and 2.3 people for *ϵ* = 2.0. ( Figure 3)

Counties displayed the same general pattern, but there are fewer counties and they typically have less empty strata, so it was not as pronounced. The size of this bias again decreased as a function of *ϵ*. Homogeneity index 0 had bias of -59.2 people for *ϵ* = 0.5, -33.9 people for *ϵ* = 1.0, and -18.8 people for **ϵ** = 2.0; while homogeneity index 22 had bias of 21.7 people for *ϵ* = 0.5, 14.5 people for *ϵ* = 1.0, and 11.1 people for *ϵ* = 2.0.

## Discussion

We anticipate some readers of this will be social researchers who rely on Census Bureau data for quantitative work, and who have concerns that the Census Bureau is going to reduce the accuracy of this data. Such a reader may be open to the possibility that privacy is a valid reason for reducing accuracy, yet still be concerned about how this will affect their next decade of research. Our results visually summarized in Figure 2 can help to understand the potential change in accuracy: if *ϵ* = 1.0, for county-level stratified counts, TopDown will be like the uncertainty introduced by working with a 50% sample of the full dataset; if *ϵ* = 2.0, it will be like working with a 75% sample; and if *ϵ* = 6.0, it will have accuracy matching a 95% sample, which is pretty close to having the full data without protecting privacy. Such a reader may still want to see an analysis like this run on the 2010 decennial census data, but we hope this will help them rest a little easier about the quality of the data they are relying on for their work.

We also expect that some readers will be more drawn to the lower end of the epsilon curve. Just how private is TopDown with *ϵ* = 0.25, especially when total count at the state-level is invariant? Our results show that all *ϵ* less than 1.0 have empirical privacy loss around 0.15, independent of *ϵ*. You can add more and more variation, but, perhaps due to the invariants, that variation does not translate into more and more privacy.

Comparing error in total count or stratified count across levels of the geographic hierarchy reveals a powerful feature of the TopDown algorithm: the error is of similar magnitude even though the counts are substantially different in size. This is because the variation added at each level has been specified to have the same portion of the total privacy budget. It remains to be investigated how alternative allocations of privacy budget across levels will change the error and empirical privacy loss.

For *ϵ* ≥ 1.0, TopDown introduced near minimal variation and attained empirical privacy loss almost 10 times less than *ϵ*. We also found that this created a quantifiable amount of bias. The bias increased the reported counts in homogeneous districts while decreasing the counts in racially and ethnically mixed districts. The TopDown algorithm may therefore drive some small amount of redistribution of resources from diverse urban communities to segregated rural communities.

Accurate counts in small communities are important for emergency preparedness and other routine planning tasks performed by state and local government demographers, and this work may help to understand how such work will be affected by the shift to a DP disclosure avoidance system.

This work has not investigated more detailed research uses of decennial census data in social research tasks, such as segregation research, and how this may be affected by TopDown.

Another important use of decennial census data is in constructing control populations and survey weights for survey sampling of the US population for health, political, and public opinion polling. Our work provides some evidence on how TopDown may affect this application, but further work is warranted.

This work fits into the beginning of a discussion on how to best balance privacy and accuracy in decennial census data collection, and there is a need for continued discussion. This need must be balanced against a risky sort of observer bias—some researchers have hypothesized that calling attention to the privacy and confidentiality of census responses, even if done in a positive manner, could reduce the willingness of respondents to answer census questions, and ongoing investigation with surveys and cognitive testing may provide some evidence on the magnitude of this effect as well as potential countermeasures ^17^.

### Limitations

There are many differences between the 1940 census data and the 2020 data to be collected next year. In addition to the US population being three times larger now, the analysis will have six geographic levels instead of four, ten times more race groups and over 60 times more age groups. We expect that this will yield detailed queries with typical precise count sizes even smaller than the stratified counts for enumeration districts we have examined here. We suspect that impact of this will likely be to slightly decrease accuracy and increase privacy loss, but the accuracy of our hypothesis remains to be seen.

In addition to the changes in the data, additional changes are planned for TopDown, such as a switch from independent geometrically distributed variation to the High Dimensional Matrix Mechanism. We expect this to increase the accuracy a small amount without changing the empirical privacy loss.

In this work, we have focused on the median of the absolute error, but the spread of this distribution is important as well, and in future work, researchers may wish to investigate the tails of this distribution. We have also focused on the empirical privacy loss for specific queries at specific geographic aggregations, and our exploration was not comprehensive. Therefore, it is possible that some other test statistic would demonstrate a larger empirical privacy loss than we have found with our approach. Our approach also assumes that the residuals for different locations in a single run are an acceptable proxy for the residuals from the same location across multiple runs. Although these are certainly different, we suspect that the difference is sufficiently small as to not affect our estimates substantially.

## Conclusion

The TopDown algorithm will provide a provably *ϵ*-DP disclosure avoidance system for the 2020 US Census, and it provides affordances to balances privacy and accuracy. This is an opportunity, but it is not without risks. Taking advantage of the opportunity and mitigating the risks will require that we understand what the approach is doing, and we hope that this analysis of the 2018 E2E test can help build such understanding.

## Data availability

### Source data

Individual-level data from the 1940 US Census is available from IPUMS https://doi.org/10.18128/D010.V8.0.EXT1940USCB ^8^.

These data are under Copyright of Minnesota Population Center, University of Minnesota. Access to the documentation is freely available without restriction; however, users must register before extracting data from the website.

The output of the TopDown algorithm when run on the 1940 US Census data is available to download from the US Census Bureau: https://www2.census.gov/census_1940/.

These data are under Copyright of the United States Census Bureau.

### Extended data

Zenodo: Extended data for Differential privacy in the 2020 US census, what will it do? Quantifying the accuracy/privacy tradeoff. https://doi.org/10.5281/zenodo.3551215 ^16^.

This project contains a full table of summary counts and errors for a range of levels of geographic hierarchy, stratification, and epsilon.

Zenodo: Supplementary Methods Appendix for Differential privacy in the 2020 US census, what will it do? Quantifying the accuracy/privacy tradeoff: Design and validation of Empirical Privacy Loss (EPL) metric. https://doi.org/10.5281/zenodo.3727242 ^15^.

This project contains additional details on the design and validation of the EPL metric used in this paper.

Extended data are available under the terms of the Creative Commons Attribution 4.0 International license (CC-BY 4.0).

### Software availability

Scripts to produce all results and figures in this paper are available online: https://github.com/aflaxman/dp_2020_census/.

Archived scripts at time of publication: https://doi.org/10.5281/zenodo.3551217 ^18^.

License: MIT License.
