# Supplementary material for: Differential privacy in the 2020 US census: what will it do? Quantifying the accuracy/privacy tradeoff
Source: Gates Open Res. 2020 Apr 6;3:1722. Originally published 2019 Dec 4. [Version 2] doi: 10.12688/gatesopenres.13089.2 (PMC7216402; doi:10.12688/gatesopenres.13089.2)
Supplement: Peer review history

# Peer review: Reviewer response for version 2

**DOI:** 10.21956/gatesopenres.14301.r28749

**Published:** 2020-05-11

**Authors:** David Van Riper

**Affiliations:** 1 IPUMS, University of Minnesota, Minneapolis, MN, USA

I am pleased with the authors' responses to my initial review. Adding extra data in the supplementary materials provides a fuller picture of the analyses they executed, and the clarifications added to the text enhance understanding. I also greatly appreciate the new Supplementary Methods Appendix that provides a more detailed discussion of the EPL measure.

Is the work clearly and accurately presented and does it cite the current literature?

Yes

If applicable, is the statistical analysis and its interpretation appropriate?

Partly

Are all the source data underlying the results available to ensure full reproducibility?

Yes

Is the study design appropriate and is the work technically sound?

Yes

Are the conclusions drawn adequately supported by the results?

Partly

Are sufficient details of methods and analysis provided to allow replication by others?

Yes

Reviewer Expertise:

geography, demography, census data, differential privacy

I confirm that I have read this submission and believe that I have an appropriate level of expertise to confirm that it is of an acceptable scientific standard.

# Peer review: Reviewer response for version 2

**DOI:** 10.21956/gatesopenres.14301.r28748

**Published:** 2020-05-06

**Authors:** Ferdinando Fioretto

**Affiliations:** 1 Syracuse University, Syracuse, NY, USA

I am happy with the authors' response to my comments and with their new revised paper. 

 While I would have liked to see a more formal analysis and description of the method evaluated, I also understand the authors’ desire to keep the article accessible to a wider audience. 

 To conclude, I believe that this article could be accepted without further revision.

Is the work clearly and accurately presented and does it cite the current literature?

Partly

If applicable, is the statistical analysis and its interpretation appropriate?

Partly

Are all the source data underlying the results available to ensure full reproducibility?

Yes

Is the study design appropriate and is the work technically sound?

Partly

Are the conclusions drawn adequately supported by the results?

Partly

Are sufficient details of methods and analysis provided to allow replication by others?

Yes

Reviewer Expertise:

Artificial Intelligence, Differential Privacy, Optimization

I confirm that I have read this submission and believe that I have an appropriate level of expertise to confirm that it is of an acceptable scientific standard.

# Peer review: Reviewer response for version 1

**DOI:** 10.21956/gatesopenres.14238.r28430

**Published:** 2020-03-03

**Authors:** Ferdinando Fioretto

**Affiliations:** 1 Syracuse University, Syracuse, NY, USA

**Overview**

 The paper examines the behavior of TopDown, a privacy-preserving algorithm proposed to release differentially private US Census data. The authors examine the privacy, accuracy, and bias trade-off induced by the application of TopDown on the 1940 US Census dataset. The analysis was detailed for various privacy loss levels (i.e., epsilon values) and compared against a simple random sampling approach. 

 The authors provide a brief overview of Differential Privacy and the TopDown algorithm. Next, they introduce the empirical privacy loss as an empirical quantification of the loss of privacy induced by the application of a differentially private mechanism, and, finally, they provide an extensive evaluation on an application of TopDown on the 1940 US Census data release.

 An interesting aspect of this work is the introduction of a novel evaluation metric, called "empirical privacy loss" or EPL. The authors argue that the use of the post-processing strategy adopted by TopDown, that projects the differentially private solution into a feasible space, may reduce the theoretical privacy loss and the experimental evaluation seem to support such claim. In particular, the authors found that the EPL for a given class of counts (total count and stratified count) is smaller than the theoretical privacy loss guaranteed by the algorithm. I have several comments about this metric, reported in the detailed comments section.

 I found this work original, in that it provides an extensive evaluation of the privacy, accuracy, and bias trade-off of the Top-Down algorithm. However, I also found the absence of a related work section unusual and would like to point out that there are other works that use optimization techniques to publish accurate count statistics, e.g.:

- Michael Hay, Vibhor Rastogi, Gerome Miklau, Dan Suciu: Boosting the Accuracy of Differentially Private Histograms Through Consistency. PVLDB 3(1): 1021-1032 (2010) ^1^.
- Chao Li, Gerome Miklau, Michael Hay, Andrew McGregor, Vibhor Rastogi: The matrix mechanism: optimizing linear counting queries under differential privacy. VLDB J. 24(6): 757-781 (2015) ^2^.

and work that pose particular emphasis on Census data: 

- Yu-Hsuan Kuo, Cho-Chun Chiu, Daniel Kifer, Michael Hay, Ashwin Machanavajjhala: Differentially Private Hierarchical Count-of-Counts Histograms. PVLDB 11(11): 1509-1521 (2018) ^3^.
- Ferdinando Fioretto, Pascal Van Hentenryck: Differential Privacy of Hierarchical Census Data: An Optimization Approach. CP 2019: 639-655 ^4^.

It may be useful to discuss some of these proposals. 

 The paper is well organized and described with a good amount of detail. However, I would have liked to see a more formal description of the TopDown algorithm and of the empirical privacy loss concept. In particular, I believe that describing TopDown using an optimization model would greatly simplify readability and avoid some doubts, such as those I list in my detailed comments. I would also suggest the authors introduce an illustration of the hierarchy utilized by the Census, together with the amount of privacy budget used at each level. This could, for instance, be visualized as a tree, where the root node describes the total counts at the national level, its children describe counts at the state level, and so on. I believe that such an illustration will ease visualizing the process performed by TopDown during Step 2, in order to satisfy the consistency of the problem constraints.  

 It would also be useful to have a table summarizing the problem constraints. For example, the authors describe equalities constraints, such as those that constrain the aggregate statistics and counts as well as those that force the invariants, and inequality constraints, such as non-negativity and properties over the group sizes. 

**Detailed Comments:**

**Section: TopDown algorithm**

- The authors provide a helpful overview of the TopDown algorithm, which operates in two steps: Noise addition and Optimization. I believe that the description can be further improved--I found the text to be quite verbose--and would encourage the authors to supply the following information:
  - A table that summarizes the attributes of the histograms to be produced (e.g., counts of each geographic by age, race, ethnicity, household/group quarters) and the aggregate statistics. 
  - An illustration highlighting the dependence between counts, and, thus, the constraints arising from these dependencies. 

I believe the above can be a helpful aid in the description of the algorithm. 

- The authors call "aggregate statistics" as "DP queries". I am not sure why this terminology was selected. At the best of my knowledge, a DP query is simply a function over a dataset that happens to satisfy DP. I would suggest using a different terminology for identifying private aggregates.
- At the end of the third paragraph of **Step One: Imprecise Histogram: **I would have preferred to see a more formal description for the computation of the histogram count and aggregate statistics. For instance, in the current version, it is not clear what is the dimensionality of each query. 
- In **Step two: Optimize:** the authors describe how TopDown optimizes the noisy estimates to satisfy the problem constraints. I would strongly suggest using a mathematical model to describe the problem (minimizer and constraints). In the current stage, a reader unfamiliar with the topic may found some sentences confusing. For example, the sentence “finds a solution that […] has the property that the value of each variable is as close as possible to the corresponding imprecise detailed histogram count or imprecise aggregate statistics” may denote that the objective is to minimize some Lp distance between the optimized counts and noisy ones; but for which p? I think that adding a formal model would improve the paper clarity. 

 
**Section: Empirical Privacy Loss **

- I found the introduction of the empirical privacy loss concept quite interesting. However, I also have a few reservations. First, I think that the formula in this section could be described in more detail. I may have missed something, but I could not find what C correspond to. Also, this formula seems to be hard to compute and I wish the authors have spent a few words on they address such a challenge. 
- The notation \hat{p}_k used in the formula \Pr[error … ] seems to have the same semantic of notation \hat{p}(x), introduced in point (2) of Section “ **Our evaluation approach**”. Is this correct, i.e., is it that \hat{p}_k = \hat{p}(k)? If this is the case, then one of the two notations need to be changed for consistency. 
- In section **TopDown Options still to be selected:**
  - On point (1): I suggest spacing the epsilon values listed;
  - On point (4): I wonder if the authors have some intuitions on why the test run used more budget for aggregated statistics than for aggregated queries. I believe it would be very insightful to discuss the implications of such budget partitioning.
- In section **Our evaluation approach**:
  - Point (2): I would have liked if the authors could have further elaborated on how the empirical privacy loss is computed. Is it the maximum among all x of ELP(x)?
  - The authors specify that the EPL is computed for the total count and they report a substantially lower loss than the theoretical privacy budget adopted. Since the privacy budget was partitioned among several levels and queries, I wonder if the authors have taken such partitioning into account when computing the final EPL score. I believe this aspect should be discussed in the text.
- Have the authors validated the fidelity of the EPL score on a simple differential privacy application? For instance, I would have liked to see a brief discussion on if this metric is in agreement with the theoretical errors provided by the Laplace mechanism on counting queries (without post-processing). 

 
**Results**

**Error and privacy of TopDown**

- The authors explain in detail the results attained in their analysis. I found the reporting of the results at the end of each subsection to be a bit distracting. I suggest the authors introduce one or multiple tables that tabulate the results and only summarize them in the text.
- Additionally, the plots in Figure 1 and the errors describes in the text are for different privacy budget: The figure illustrates the errors for epsilon = 0.5, 1.0, and 2.0, while the text describes the errors for epsilon = 0.25, 1.0, and 4.0. I suggest the authors reporting the results for all the epsilon tested into a table, or to make the description in the text and the figure consistent for the privacy budgets adopted.
- The empirical privacy loss computed was reported for the total count at the enumeration district level and country-level and compared against the privacy budget adopted by the TopDown algorithm. As stated in my comment above, I wonder if this comparison is fair. TopDown seems to partition the privacy budget for different queries, thus leaving the total count queries with substantially less budget than the original total one. I encourage the author to expand on this aspect of the evaluation.

**Comparison with error and privacy of simple random sampling**

- As for the previous section, I recommend the authors to use a table to tabulate the numerical results described in the last paragraph. In my opinion, it will substantially increase readability. 

**Bias in the variation introduced by TopDown**

- As for the previous section, I suggest the authors tabulate the results of the homogeneity index and bias. 
- Are the errors by homogeneity index an average over the sample runs?

Is the work clearly and accurately presented and does it cite the current literature?

Partly

If applicable, is the statistical analysis and its interpretation appropriate?

Partly

Are all the source data underlying the results available to ensure full reproducibility?

Yes

Is the study design appropriate and is the work technically sound?

Partly

Are the conclusions drawn adequately supported by the results?

Partly

Are sufficient details of methods and analysis provided to allow replication by others?

Yes

Reviewer Expertise:

Artificial Intelligence, Differential Privacy, Optimization

I confirm that I have read this submission and believe that I have an appropriate level of expertise to confirm that it is of an acceptable scientific standard, however I have significant reservations, as outlined above.

## References

[1] : Boosting the accuracy of differentially private histograms through consistency. *Proceedings of the VLDB Endowment*.2010;3(1-2) : 10.14778/1920841.1920970 1021-1032 10.14778/1920841.1920970

[2] : The matrix mechanism: optimizing linear counting queries under differential privacy. *The VLDB Journal*.2015;24(6) : 10.1007/s00778-015-0398-x 757-781 10.1007/s00778-015-0398-x

[3] : Differentially private hierarchical count-of-counts histograms. *Proceedings of the VLDB Endowment*.2018;11(11) : 10.14778/3236187.3236202 1509-1521 10.14778/3236187.3236202

[4] : Differential Privacy of Hierarchical Census Data: An Optimization Approach.2019;11802: 10.1007/978-3-030-30048-7_37 639-655 10.1007/978-3-030-30048-7_37

# Response

**Published:** 2020-03-20

**Authors:** Abraham Flaxman

**Affiliations:** University of Washington, Seattle, USA

**Overview**

 The paper examines the behavior of TopDown, a privacy-preserving algorithm proposed to release differentially private US Census data. The authors examine the privacy, accuracy, and bias trade-off induced by the application of TopDown on the 1940 US Census dataset. The analysis was detailed for various privacy loss levels (i.e., epsilon values) and compared against a simple random sampling approach. 

 The authors provide a brief overview of Differential Privacy and the TopDown algorithm. Next, they introduce the empirical privacy loss as an empirical quantification of the loss of privacy induced by the application of a differentially private mechanism, and, finally, they provide an extensive evaluation on an application of TopDown on the 1940 US Census data release.

 An interesting aspect of this work is the introduction of a novel evaluation metric, called "empirical privacy loss" or EPL. The authors argue that the use of the post-processing strategy adopted by TopDown, that projects the differentially private solution into a feasible space, may reduce the theoretical privacy loss and the experimental evaluation seem to support such claim. In particular, the authors found that the EPL for a given class of counts (total count and stratified count) is smaller than the theoretical privacy loss guaranteed by the algorithm. I have several comments about this metric, reported in the detailed comments section.

 I found this work original, in that it provides an extensive evaluation of the privacy, accuracy, and bias trade-off of the Top-Down algorithm. However, I also found the absence of a related work section unusual and would like to point out that there are other works that use optimization techniques to publish accurate count statistics, e.g.:

- Michael Hay, Vibhor Rastogi, Gerome Miklau, Dan Suciu: Boosting the Accuracy of Differentially Private Histograms Through Consistency. PVLDB 3(1): 1021-1032 (2010) ^1^.
- Chao Li, Gerome Miklau, Michael Hay, Andrew McGregor, Vibhor Rastogi: The matrix mechanism: optimizing linear counting queries under differential privacy. VLDB J. 24(6): 757-781 (2015) ^2^.

and work that pose particular emphasis on Census data: 

- Yu-Hsuan Kuo, Cho-Chun Chiu, Daniel Kifer, Michael Hay, Ashwin Machanavajjhala: Differentially Private Hierarchical Count-of-Counts Histograms. PVLDB 11(11): 1509-1521 (2018) ^3^.
- Ferdinando Fioretto, Pascal Van Hentenryck: Differential Privacy of Hierarchical Census Data: An Optimization Approach. CP 2019: 639-655 ^4^.

It may be useful to discuss some of these proposals. 

*We thank the reviewer for this cogent assessment of our work, as well as for identifying these additional relevant references on the use of optimization techniques in producing differentially private histograms. We have added references to these in our methods section, following the description of the TopDown algorithm.*

 The paper is well organized and described with a good amount of detail. However, I would have liked to see a more formal description of the TopDown algorithm and of the empirical privacy loss concept. In particular, I believe that describing TopDown using an optimization model would greatly simplify readability and avoid some doubts, such as those I list in my detailed comments. I would also suggest the authors introduce an illustration of the hierarchy utilized by the Census, together with the amount of privacy budget used at each level. This could, for instance, be visualized as a tree, where the root node describes the total counts at the national level, its children describe counts at the state level, and so on. I believe that such an illustration will ease visualizing the process performed by TopDown during Step 2, in order to satisfy the consistency of the problem constraints.  

 It would also be useful to have a table summarizing the problem constraints. For example, the authors describe equalities constraints, such as those that constrain the aggregate statistics and counts as well as those that force the invariants, and inequality constraints, such as non-negativity and properties over the group sizes. 

*We appreciate the reviewer’s suggestion that we include more precise technical details about the optimization model and the geographic hierarchy use by Census, but we would prefer not to over-burden our anticipated readership with this level of detail.  Our goal is to give a detailed overview, but only an overview, not a definitive source of the details of TopDown.  The Census Bureau has released their own technical documentation of their system (e.g. reference [9] of our text) as well as their source code, and readers who want to get the full details from the definitive source should go to the papers and code from Census for this.  Also, the specifics of the geographic hierarchy and the allocation of epsilon between its levels is still work-in-progress at Census Bureau, and while we know precisely what they did for the 2018 end-to-end test, we do expect this to change as they finish the implementation and parameter selection process for the analyzing the 2020 decennial census.*

**Detailed Comments:**

**Section: TopDown algorithm**

- The authors provide a helpful overview of the TopDown algorithm, which operates in two steps: Noise addition and Optimization. I believe that the description can be further improved--I found the text to be quite verbose--and would encourage the authors to supply the following information:
  - A table that summarizes the attributes of the histograms to be produced (e.g., counts of each geographic by age, race, ethnicity, household/group quarters) and the aggregate statistics. 
  - An illustration highlighting the dependence between counts, and, thus, the constraints arising from these dependencies. 

I believe the above can be a helpful aid in the description of the algorithm. 

*As we responded above, we appreciate the reviewer’s suggestion to include more precise technical details about the detailed histogram and the aggregate statistics, and the dependencies between the counts, but as we responded above, we believe that these are all still work-in-progress for the 2020 census, and do not want to draw too much attention to the test values that were used in the 2018 end-to-end test. *

- The authors call "aggregate statistics" as "DP queries". I am not sure why this terminology was selected. At the best of my knowledge, a DP query is simply a function over a dataset that happens to satisfy DP. I would suggest using a different terminology for identifying private aggregates.

*We initially planned to use the term “aggregate statistic” but decided that since the Census Bureau has used the term “DP query” internally, it would be helpful to give our readers this jargon in case they wanted to advocate for allocating privacy budget to specific DP queries that are relevant to their uses of census data.* *We have* *edited the text where this jargon is first introduced to better identify it as Census Bureau terminology.* 

- At the end of the third paragraph of **Step One: Imprecise Histogram: **I would have preferred to see a more formal description for the computation of the histogram count and aggregate statistics. For instance, in the current version, it is not clear what is the dimensionality of each query. 

*We appreciate the reviewer’s desire for more formal descriptions and more precision in the methods section of this paper, and we regret if our responses are beginning to become repetitive, but we prefer to keep things from becoming *overly formal, as our goal is to reach an audience that has not yet been reached by the more “computer science-y” publications from the Census Bureau that have described their approach. To the reviewer’s example of the dimensionality of each query, there are 2*6*2*6 = 144 counts for each geographic area (the counts are stratified by age (two values: under-18-year-olds and 18-plus), race (six values), ethnicity (two values: Hispanic and non-Hispanic), and household/group-quarters type (6 values)).

- In **Step two: Optimize:** the authors describe how TopDown optimizes the noisy estimates to satisfy the problem constraints. I would strongly suggest using a mathematical model to describe the problem (minimizer and constraints). In the current stage, a reader unfamiliar with the topic may found some sentences confusing. For example, the sentence “finds a solution that […] has the property that the value of each variable is as close as possible to the corresponding imprecise detailed histogram count or imprecise aggregate statistics” may denote that the objective is to minimize some Lp distance between the optimized counts and noisy ones; but for which p? I think that adding a formal model would improve the paper clarity. 

*We have adjusted this language to make it clearer that this is a quadratic objective function, i.e. Lp for p = 2. As above, we want to avoid writing out the constrained convex optimization problem in mathematical notation, because we anticipate a substantial portion of our target readership will prefer *not *to see it in this form, although we appreciate that there are also those, like the reviewer, who *will *want to see it in this form.*

**Section: Empirical Privacy Loss **

- I found the introduction of the empirical privacy loss concept quite interesting. However, I also have a few reservations. First, I think that the formula in this section could be described in more detail. I may have missed something, but I could not find what C correspond to. Also, this formula seems to be hard to compute and I wish the authors have spent a few words on they address such a challenge. 

*We have added supplementary material that provides substantial additional detail on how EPL is defined and how it works.*


*  *

- The notation \hat{p}_k used in the formula \Pr[error … ] seems to have the same semantic of notation \hat{p}(x), introduced in point (2) of Section “ **Our evaluation approach**”. Is this correct, i.e., is it that \hat{p}_k = \hat{p}(k)? If this is the case, then one of the two notations need to be changed for consistency. 

*We have improved this notation to accompany our improved exposition of EPL in the main text and supplementary material.*


*  *

- In section **TopDown Options still to be selected:**
  - On point (1): I suggest spacing the epsilon values listed;

*Good idea, done.*

  

- On point (4): I wonder if the authors have some intuitions on why the test run used more budget for aggregated statistics than for aggregated queries. I believe it would be very insightful to discuss the implications of such budget partitioning.

*There is no source of information we know of that justifies the choice Census Bureau made for splitting the privacy budget between the detailed queries and DP queries.  We agree that an improved understanding of the tradeoffs between different budgets is an important direction for future research, but we feel that it is beyond the scope of the present paper.*

  

- In section **Our evaluation approach**:
  - Point (2): I would have liked if the authors could have further elaborated on how the empirical privacy loss is computed. Is it the maximum among all x of ELP(x)?

*We apologize for the lack of detail in our paper and have added more precision in the main text as well as supplementary material with more detail.  The scalar *EPL *is the maximum of the absolute value of *EPL( *x*) over all *x.*

  

- The authors specify that the EPL is computed for the total count and they report a substantially lower loss than the theoretical privacy budget adopted. Since the privacy budget was partitioned among several levels and queries, I wonder if the authors have taken such partitioning into account when computing the final EPL score. I believe this aspect should be discussed in the text.

*We intend the EPL to be compared with the overall epsilon, and therefore not to require any consideration of how the privacy budget was partitioned.  Indeed, we want to be able to compute EPL for algorithms that are *not *provably differentially private, or for algorithms that use many levels or no levels. Perhaps we are misunderstanding the reviewer’s question/suggestion.*


*  *

- Have the authors validated the fidelity of the EPL score on a simple differential privacy application? For instance, I would have liked to see a brief discussion on if this metric is in agreement with the theoretical errors provided by the Laplace mechanism on counting queries (without post-processing). 

*We hope that the first example from the new supplementary material will address exactly this request, by calculating the EPL of a Geometric Mechanism with epsilon=0.25.  Our Supplementary Appendix Table SA1 also includes a table of EPL values for the Geometric Mechanism a range of epsilon values and random seeds, and provides some evidence that EPL works in this setting:* 

**(a) epsilon value in Geometric DP **EPL mean EPL Lower Bound (2.5th Percentile) EPL Upper Bound (97.5th Percentile)

 eps       EPL     lower   upper

 0.0010 0.0010 0.0008 0.0013

**0.0050** 0.0048 0.0039 0.0068

**0.0100 **0.0099 0.0076 0.0130

**0.0500 **0.0490 0.0390 0.0673

**0.1000** 0.0980 0.0752 0.1262

**0.1500** 0.1475 0.1181 0.1941

**0.2000 **0.1988 0.1521 0.2639

**Results**

**Error and privacy of TopDown**

- The authors explain in detail the results attained in their analysis. I found the reporting of the results at the end of each subsection to be a bit distracting. I suggest the authors introduce one or multiple tables that tabulate the results and only summarize them in the text.

*We appreciate this suggestion, but we also want to reach audiences that prefer figures and text, as well as those who like tables. We do have a table of all results as supplementary material, and we have added an additional table with results for the SRS comparisons.*

- Additionally, the plots in Figure 1 and the errors describes in the text are for different privacy budget: The figure illustrates the errors for epsilon = 0.5, 1.0, and 2.0, while the text describes the errors for epsilon = 0.25, 1.0, and 4.0. I suggest the authors reporting the results for all the epsilon tested into a table, or to make the description in the text and the figure consistent for the privacy budgets adopted.

*Thank you for this suggestion.  We have updated the text to refer to epsilon values that match the figures.  As mentioned above, we also have included values for all epsilons in our supplementary materials.*

- The empirical privacy loss computed was reported for the total count at the enumeration district level and country-level and compared against the privacy budget adopted by the TopDown algorithm. As stated in my comment above, I wonder if this comparison is fair. TopDown seems to partition the privacy budget for different queries, thus leaving the total count queries with substantially less budget than the original total one. I encourage the author to expand on this aspect of the evaluation.

*We prioritized total count in our figures because of its importance and simplicity, but in our supplementary tables, we also include EPL for counts stratified by age group/race/ethnicity. In the 2018 end-to-end test, there is substantially less stratification than there will be in the 2020 decennial census, and this will be an important area for further research with more recent data.*

**Comparison with error and privacy of simple random sampling**

- As for the previous section, I recommend the authors to use a table to tabulate the numerical results described in the last paragraph. In my opinion, it will substantially increase readability. 

*We appreciate this suggestion, but we also want to reach audiences that prefer figures and text, as well as those who like tables. We do have a table of all results as supplementary material, and we have added an additional table with results for the SRS comparisons.*

**Bias in the variation introduced by TopDown**

- As for the previous section, I suggest the authors tabulate the results of the homogeneity index and bias. 

*We don’t have a table for this, and we are hesitant to put one in. We think that the figure provides a clearer communication of the pattern (mean residual goes up and to the right as a function of homogeneity index, but the change is attenuated as epsilon gets larger) and the precise numbers are available in the text for any reader who wants this level of precision. Although we greatly appreciate the reviewer’s work in commenting on our paper, we have come to see that we just do not value tables of results the way the reviewer does. We hope that this difference in presentation styles does not stand in the way of a producing a strong and scientifically valid paper.*

  

- Are the errors by homogeneity index an average over the sample runs?

*The distributions are for errors pooled over the four random seeds (for each epsilon/homogeneity index), and the markers are the average bias across all four runs. We have added to the caption of the figure to make this clear.*

- Is the work clearly and accurately presented and does it cite the current literature?

Partly

- Is the study design appropriate and is the work technically sound?

Partly

- Are sufficient details of methods and analysis provided to allow replication by others?

Yes

- If applicable, is the statistical analysis and its interpretation appropriate?

Partly

- Are all the source data underlying the results available to ensure full reproducibility?

Yes

- Are the conclusions drawn adequately supported by the results?

Partly

*Thank you for your work reviewing this paper!*

# Peer review: Reviewer response for version 1

**DOI:** 10.21956/gatesopenres.14238.r28334

**Published:** 2019-12-20

**Authors:** David Van Riper

**Affiliations:** 1 IPUMS, University of Minnesota, Minneapolis, MN, USA

**Overview**

 Using differentially private 1940 census data produced by the US Census Bureau's TopDown algorithm, Petti and Flaxman assess the privacy/accuracy trade-off along multiple dimensions for this algorithm for multiple values of epsilon. The authors analyzed the median absolute error, empirical privacy loss, and bias for the differentially private data. They also compared the median absolute error and empirical privacy loss for differentially private data with data generated through simple random sampling. This is one of the first, if not the first, article assessing the accuracy of decennial census data published through a differentially private algorithm.

 Petti and Flaxman provide a good overview of differential privacy and the Census Bureau's TopDown algorithm - a differentially private algorithm for producing decennial census data. They then compare the differentially private 1940 data with the original complete-count 1940 data to assess the accuracy introduce by the TopDown algorithm. They find that error increased as the total privacy loss budget decreased. They also find that empirical privacy loss was smaller than total privacy loss budget. They measure bias introduced by the algorithm and find that bias increases as homogeneity decreases and that bias increases as total privacy loss budget decreases. They conclude that privacy loss does not vary much for epsilon < 1.0, and that the accuracy achieved when using a 50% simple random sample is equivalent to an epsilon of 1.0.

 I am intrigued by the empirical privacy loss measure introduced by Petti and Flaxman. Its formula and interpretation mirrors the formula for epsilon-differential privacy. However, I would like to see a more thorough discussion of empirical privacy loss summary statistic reported in the results section of the paper. The authors compare an empirical privacy loss summary statistic with total privacy loss budget on pages 6 and 7 of the paper, but they never explain how the summary statistic was computed. Having that explanation would help me better understand the comparison they make throughout the paper.

 The authors compare the empirical privacy loss for a given geographic unit-type of count (total count, stratified count) combination with the overall privacy loss budget. They empirical privacy loss for a given combination is less than the overall privacy loss budget. I wonder if this is the correct comparison to make. The privacy loss budget controls the overall amount of privacy leaked by the publication of all statistics. It is the sum, via sequential composition, of the epsilon fractions assigned to each geographic level-statistic combination. Thus, by definition, the empirical privacy loss associated with a particular geographic level-statistic (e.g., total population count) must be less than the privacy loss budget. I would like to see a fuller discussion of this comparison in the paper. See detailed comment #14 for more details. 

 I would also additional supplemental datasets (or tables in the paper) with the empirical privacy loss summary statistics for all values of epsilon. The authors report a few values in the text and figures, but having a complete set would allow for a more comprehensive understanding of the relationship between empirical privacy loss and epsilon.

 Finally, I strongly recommend that the authors use the same examples in their text as they use in the figures. The text uses epsilons of 0.25, 1.0 and 4.0 and the figures use epsilons of 0.50, 1.0, and 2.0. Making the epsilons consistent between the text and figures will help the reader better understand the analysis. 

***Detailed comments by section***

**Methods - TopDown algorithm**

- The authors' high level overview (first paragraph in subsection entitled "TopDown algorithm") describe the noise injection (Imprecise Histogram) and optimization steps in the TopDown algorithm. They state that the "second step (Optimize) adjusts the histogram to be close as possible to the imprecise counts". I am uncertain about what histogram the authors refer to in this sentence. Is the histogram based on the original data, or is this the noise-injected detailed histogram? My understanding of the algorithm is that is generates histograms (one for each combination of geographic level and query) from the original data and then injects noise into histograms using the appropriate two-sided geometric distribution. It then passes these noise-injected histograms to the optimization function.

I would like the authors to be more precise in their description of the histogram and the "imprecise counts" in this section.

- The authors state that the 2020 US Census will have six geographic levels nested hierarchically (last sentence of TopDown algorithm paragraph). The Census Bureau allocated privacy loss budget to seven nested geographies (nation, state, county, tract group, census tract, block group, block) for the 2010 demonstration product. The Bureau has not committed to this allocation for 2020 and could still change the allocation strategy. I recommend clarifying that statement to pertain solely to the 2010 demonstration data product.
- In the final clause of the last sentence of the TopDown algorighm paragraph, the authors state that "in the 1940 E2E test, only national, state, county, and district levels were included." I recommend adding the word "enumeration" before district in that clause.

**Methods - Step one: Imprecise Histogram**

- At the end of first paragraph in this section, the authors describe the "ethnicity-age" aggregate statistic set. The implication of this sentence is that the "ethnicity-age" aggregate statistics set was one pre-selected by Census for noise injection. Census did not choose this aggregate statistic set. The aggregate statistic sets chosen by census were Voting age by Hispanic origin by Race (a 2 x 2 x 6 cell query) and Household/Group quarter (a 6 cell query). I recommend modifying this sentence to describe one of the two pre-selected aggregate statistic sets.
- At the end of the second paragraph, the authors write that "22.5% spent on the group-quarters queries". I recommend changing the fragment to be "22.5% spent on the household/group-quarters queries". The word "household" is important when discussing this DP query. People can either live in household or group quarters, and by definition, households are not group quarters.

**Methods - TopDown options still to be selected**

- For option 3, I recommend modifying the "(and therefore aggregated over "group quarters types)" to be "(there therefore aggregated over "household/group quarters types)".  A household is not a type of group quarter.
- Also in option 3, I recommend modifying the "(ii) the group-quarters counts" to be "(ii) the household/group quarters counts".
- In option 5, add the word "population" between "total" and "count" in the second sentence. Otherwise, readers will not necessarily know which total count to which the authors are referring.

**Results - Error and privacy of TopDown**

- At the end of first paragraph of this subsection, the authors list the median and 95th percentile of TC for EDs, counties, and states. I think it is important to clarify that these counts are based on the original 1940 census data and not on any of the differentially private 1940 datasets. Since this sentence comes at the end of a paragraph describing median absolute error, readers may assume the medians and 95th percentiles are from a DP dataset. Consider moving that sentence up the start of the paragraph.
- At the end of second paragraph of this subsection, the authors list the median and 95th percentile of SC for EDs, counties, and states. I think it is important to clarify that these counts are based on the original 1940 census data and not on any of the differentially private 1940 datasets. Since this sentence comes at the end of a paragraph describing median absolute error, readers may assume the medians and 95th percentiles are from a DP dataset. Consider moving that sentence up the start of the paragraph.
- The final two paragraphs of this subsection describe the empirical privacy loss for TC and SC for different geographic levels and different epsilons. They describe the EPL for epsilons of 0.25, 1.0, and 4.0 in the text. I would like to have a table, either in the paper or in the extended data product, that lists the EPLs for all values of epsilon and all geographic levels for TC and SC. I wonder how linear the relationship between EPL and epsilon is.  
- The authors list a number of EPL values in the final two paragraphs and in the right-hand panel of Figure 1, but I do not know what the EPL value represents. Is it the absolute value of the maximum observed EPL, or is it the range from the maximum to minimum observed EPL value? I would appreciate a more complete discussion of how the authors calculated the value of EPL they plot in Figure 1 and list in the text. The formula on page 5 describes how to compute EPL for a single geographic unit and value of epsilon, but I don't see how that formula extends to the summary statistics reported on page 6.
- Figure 1 plots the error and EPL for epsilon equal to 0.5, 1.0, and 2.0, but the text in the final two paragraphs describes EPL for epsilons of 0.25, 1.0, and 4.0. I strongly recommend making the values in the text and the plot consistent with one another. That consistency will make it easier to interpret the plot in Figure 1.
- The authors compare the empirical privacy loss for a given geographic unit-type of count (total count, stratified count) combination with the overall privacy loss budget. They empirical privacy loss for a given combination is less than the overall privacy loss budget. I wonder if this is the correct comparison to make. The privacy loss budget controls the overall amount of privacy leaked by the publication of all statistics. It is the sum, via sequential composition, of the epsilon fractions assigned to each geographic level-statistic combination. Thus, by definition, the empirical privacy loss associated with a particular geographic level-statistic (e.g., total population count) must be less than the privacy loss budget.

For a given value of epsilon, we can compute the portion of that value that is assigned to each geographic level - query combination. For example, epsilon of 0.25 is divided up as follows:

 Geographic levels = 0.25 to each level

 Tables = 0.1 (detailed), 0.225 (household-group quarters), 0.675 (voting age - Hispanic - race)

 We can multiply the geographic level fraction by the table fractions by epsilon to yield:

 Geog level - detailed query = 0.00625 epsilon

 Geog level - household group quarters query = 0.0140625 epsilon

 Geog level - voting age - Hispanic - race query = 0.0421875 epsilon

 These epsilons still do not equate to an epsilon associated with a particular statistic, such as total population count. Given the optimization step and the state-level total population invariant, I'm not sure if we can compute an epsilon value for a particular statistic. But these epsilon values seems like a more appropriate comparison to the empirical privacy loss reported by the authors.

**Results - Comparison with error and privacy of simple random sampling**

- I would like to have a table of MAE and EPL values for Simple Random Sampling. Consider adding those values to the Extended Data product currently available, or adding another Extended Data product with these values.
- Consider adding a plot of EPL by sample size to supplement or even replace the final paragraph of this subsection. There are a lot of numbers in the final paragraph, and I find it difficult to visualize the relationship between EPL and sampling fraction just by reading the numbers.
- The x-axis for Figure 2 depicts values of Empirical Privacy Loss, but neither the text nor the caption describe how the values were computed. This comment fits with comment 12 - what does the Empirical Privacy Loss summary statistic mean and how was it computed.

**Results - Bias in the variation introduced by TopDown**

- Figure 3 plots the error and EPL for epsilon equal to 0.5, 1.0, and 2.0, but the text in the first paragraph describes EPL by homogeneity index for epsilons of 0.25, 1.0, and 4.0. I strongly recommend making the values in the text and the plot consistent with one another. That consistency will make it easier to interpret the plot in Figure 3.
- I recommend moving the (Figure 3) parenthetical to the end of the discussion on EPL by homogeneity for enumeration districts. Figure 3 only shows the results for enumeration districts, but the parenthetical comes after the discussion for counties.
- In the paragraph and Figure 3, the authors list a summary statistic for bias by homogeneity index and epsilon. Is the summary statistic the mean or the median?
- Figure 3 displays the violin plot/mean bias for 11 of 23 homogeneity index values. I recommend modifying the figure caption to indicate that the authors are only displaying some of the homogeneity index values on the plot.
- I also recommend modifying the x-axis label to indicate that the homogeneity index values are for enumeration districts. That would help readers immediately understand what geographic units are being plotted.

Is the work clearly and accurately presented and does it cite the current literature?

Yes

If applicable, is the statistical analysis and its interpretation appropriate?

Partly

Are all the source data underlying the results available to ensure full reproducibility?

Yes

Is the study design appropriate and is the work technically sound?

Yes

Are the conclusions drawn adequately supported by the results?

Partly

Are sufficient details of methods and analysis provided to allow replication by others?

Yes

Reviewer Expertise:

geography, demography, census data, differential privacy

I confirm that I have read this submission and believe that I have an appropriate level of expertise to confirm that it is of an acceptable scientific standard, however I have significant reservations, as outlined above.

# Response

**Published:** 2020-03-20

**Authors:** Abraham Flaxman

**Affiliations:** University of Washington, Seattle, USA

**Reviewer 1 Feedback from David Van Riper**

**Overview**

 Using differentially private 1940 census data produced by the US Census Bureau's TopDown algorithm, Petti and Flaxman assess the privacy/accuracy trade-off along multiple dimensions for this algorithm for multiple values of epsilon. The authors analyzed the median absolute error, empirical privacy loss, and bias for the differentially private data. They also compared the median absolute error and empirical privacy loss for differentially private data with data generated through simple random sampling. This is one of the first, if not the first, article assessing the accuracy of decennial census data published through a differentially private algorithm.

 Petti and Flaxman provide a good overview of differential privacy and the Census Bureau's TopDown algorithm - a differentially private algorithm for producing decennial census data. They then compare the differentially private 1940 data with the original complete-count 1940 data to assess the accuracy introduce by the TopDown algorithm. They find that error increased as the total privacy loss budget decreased. They also find that empirical privacy loss was smaller than total privacy loss budget. They measure bias introduced by the algorithm and find that bias increases as homogeneity decreases and that bias increases as total privacy loss budget decreases. They conclude that privacy loss does not vary much for epsilon < 1.0, and that the accuracy achieved when using a 50% simple random sample is equivalent to an epsilon of 1.0.

 I am intrigued by the empirical privacy loss measure introduced by Petti and Flaxman. Its formula and interpretation mirrors the formula for epsilon-differential privacy. However, I would like to see a more thorough discussion of empirical privacy loss summary statistic reported in the results section of the paper. The authors compare an empirical privacy loss summary statistic with total privacy loss budget on pages 6 and 7 of the paper, but they never explain how the summary statistic was computed. Having that explanation would help me better understand the comparison they make throughout the paper.

*Thank you for your interest in this construct. We have added a substantially more detailed exposition of EPL in a Supplementary Methods Appendix.*

 The authors compare the empirical privacy loss for a given geographic unit-type of count (total count, stratified count) combination with the overall privacy loss budget. The empirical privacy loss for a given combination is less than the overall privacy loss budget. I wonder if this is the correct comparison to make. The privacy loss budget controls the overall amount of privacy leaked by the publication of all statistics. It is the sum, via sequential composition, of the epsilon fractions assigned to each geographic level-statistic combination. Thus, by definition, the empirical privacy loss associated with a particular geographic level-statistic (e.g., total population count) must be less than the privacy loss budget. I would like to see a fuller discussion of this comparison in the paper. See detailed comment #14 for more details. 

*In any epsilon-DP algorithm, we expect that the EPL that we have defined will be less than the privacy loss budget overall. However, there is not a formal guarantee that the privacy loss at a geographic level of TopDown will be less than the overall epsilon (due to invariants), and we believe that it is useful to see that EPL suggests that in practice the privacy loss at non-invariant levels is lower than the overall epsilon.  Even for a DP version, such as TopDown with no invariants, although it is logical to suspect that the inequality from sequential composition has slack, this is also not guaranteed. We have tried to compare some of these claims in simplified settings in the supplementary material we have added about EPL.*

 I would also additional supplemental datasets (or tables in the paper) with the empirical privacy loss summary statistics for all values of epsilon. The authors report a few values in the text and figures, but having a complete set would allow for a more comprehensive understanding of the relationship between empirical privacy loss and epsilon.

*We have added an EPL column to the table in the supplementary appendix.*

 Finally, I strongly recommend that the authors use the same examples in their text as they use in the figures. The text uses epsilons of 0.25, 1.0 and 4.0 and the figures use epsilons of 0.50, 1.0, and 2.0. Making the epsilons consistent between the text and figures will help the reader better understand the analysis. 

*Thank you for this feedback.  We have harmonized the text and figures to all use the epsilon values of 0.5, 1.0, and 2.0, because these provide a clearer visual comparison and epsilon 2.0 produces a noise level in finest-grained detailed queries most similar to that used in the census tract detailed queries of the 2010 demonstration product.*

***Detailed comments by section***

**Methods - TopDown algorithm**

- The authors' high level overview (first paragraph in subsection entitled "TopDown algorithm") describe the noise injection (Imprecise Histogram) and optimization steps in the TopDown algorithm. They state that the "second step (Optimize) adjusts the histogram to be close as possible to the imprecise counts". I am uncertain about what histogram the authors refer to in this sentence. Is the histogram based on the original data, or is this the noise-injected detailed histogram? My understanding of the algorithm is that is generates histograms (one for each combination of geographic level and query) from the original data and then injects noise into histograms using the appropriate two-sided geometric distribution. It then passes these noise-injected histograms to the optimization function.
  I would like the authors to be more precise in their description of the histogram and the "imprecise counts" in this section.

*The reviewer’s understanding matches with ours, and we have edited the phrasing in this paragraph to try to make this clearer.*

- The authors state that the 2020 US Census will have six geographic levels nested hierarchically (last sentence of TopDown algorithm paragraph). The Census Bureau allocated privacy loss budget to seven nested geographies (nation, state, county, tract group, census tract, block group, block) for the 2010 demonstration product. The Bureau has not committed to this allocation for 2020 and could still change the allocation strategy. I recommend clarifying that statement to pertain solely to the 2010 demonstration data product.

*We have backed off this claim, by changing “will” to “may”. At this point, it seems possible that the hierarchy will even be substantially different than what was used in the public demonstrations so far.*


*  *

- In the final clause of the last sentence of the TopDown algorithm paragraph, the authors state that "in the 1940 E2E test, only national, state, county, and district levels were included." I recommend adding the word "enumeration" before district in that clause.

*Added.*

**Methods - Step one: Imprecise Histogram**

- At the end of first paragraph in this section, the authors describe the "ethnicity-age" aggregate statistic set. The implication of this sentence is that the "ethnicity-age" aggregate statistics set was one pre-selected by Census for noise injection. Census did not choose this aggregate statistic set. The aggregate statistic sets chosen by census were Voting age by Hispanic origin by Race (a 2 x 2 x 6 cell query) and Household/Group quarter (a 6 cell query). I recommend modifying this sentence to describe one of the two pre-selected aggregate statistic sets.

*Good idea.*


*  *

- At the end of the second paragraph, the authors write that "22.5% spent on the group-quarters queries". I recommend changing the fragment to be "22.5% spent on the household/group-quarters queries". The word "household" is important when discussing this DP query. People can either live in household or group quarters, and by definition, households are not group quarters.

*Good point.  We have also propagated this change through the rest of the document.*

**Methods - TopDown options still to be selected**

- For option 3, I recommend modifying the "(and therefore aggregated over "group quarters types)" to be "(there therefore aggregated over "household/group quarters types)".  A household is not a type of group quarter.

*Agree.*


*  *

- Also in option 3, I recommend modifying the "(ii) the group-quarters counts" to be "(ii) the household/group quarters counts".

*Agree.*


*  *

- In option 5, add the word "population" between "total" and "count" in the second sentence. Otherwise, readers will not necessarily know which total count to which the authors are referring.

*Agree, good point.*

**Results - Error and privacy of TopDown**

- At the end of first paragraph of this subsection, the authors list the median and 95th percentile of TC for EDs, counties, and states. I think it is important to clarify that these counts are based on the original 1940 census data and not on any of the differentially private 1940 datasets. Since this sentence comes at the end of a paragraph describing median absolute error, readers may assume the medians and 95th percentiles are from a DP dataset. Consider moving that sentence up the start of the paragraph.

*We have attempted to clarify this without restructuring the paragraph, and also added a similar clarification to the methods section.*


*  *

- At the end of second paragraph of this subsection, the authors list the median and 95th percentile of SC for EDs, counties, and states. I think it is important to clarify that these counts are based on the original 1940 census data and not on any of the differentially private 1940 datasets. Since this sentence comes at the end of a paragraph describing median absolute error, readers may assume the medians and 95th percentiles are from a DP dataset. Consider moving that sentence up the start of the paragraph.

*We have attempted to clarify this without restructuring the paragraph, and also added a similar clarification to the methods section.*

**

  

- The final two paragraphs of this subsection describe the empirical privacy loss for TC and SC for different geographic levels and different epsilons. They describe the EPL for epsilons of 0.25, 1.0, and 4.0 in the text. I would like to have a table, either in the paper or in the extended data product, that lists the EPLs for all values of epsilon and all geographic levels for TC and SC. I wonder how linear the relationship between EPL and epsilon is.  

*Good idea.  There is a table of extended data already, but we failed to include a column of EPL data in it! We have added this column, and since your wondering got us wondering, we examined this relationship and found that it goes relatively linearly up-and-to-the-right.  Unfortunately, we cannot figure out how to include this figure in our response.*

  

- The authors list a number of EPL values in the final two paragraphs and in the right-hand panel of Figure 1, but I do not know what the EPL value represents. Is it the absolute value of the maximum observed EPL, or is it the range from the maximum to minimum observed EPL value? I would appreciate a more complete discussion of how the authors calculated the value of EPL they plot in Figure 1 and list in the text. The formula on page 5 describes how to compute EPL for a single geographic unit and value of epsilon, but I don't see how that formula extends to the summary statistics reported on page 6.

*We have added supplementary material with a more detailed exposition of the EPL value, as well as including a short, but more precise definition in the methods section.  However, some busy readers are sure to look first at the figures, so we have also attempted to clarify here that the EPL value is the maximum of the absolute value of the log of p^KDE(x) / p^KDE(x+1)* *over all x* *.*

  

- Figure 1 plots the error and EPL for epsilon equal to 0.5, 1.0, and 2.0, but the text in the final two paragraphs describes EPL for epsilons of 0.25, 1.0, and 4.0. I strongly recommend making the values in the text and the plot consistent with one another. That consistency will make it easier to interpret the plot in Figure 1.

*Thank you for this feedback.  We have harmonized the text and figures to all use the epsilon values of 0.5, 1.0, and 2.0, because these provide a clearer visual comparison and epsilon 2.0 produces a noise level in finest-grained detailed queries most similar to that used in the census tract detailed queries of the 2010 demonstration product.*

  

- The authors compare the empirical privacy loss for a given geographic unit-type of count (total count, stratified count) combination with the overall privacy loss budget. They empirical privacy loss for a given combination is less than the overall privacy loss budget. I wonder if this is the correct comparison to make. The privacy loss budget controls the overall amount of privacy leaked by the publication of all statistics. It is the sum, via sequential composition, of the epsilon fractions assigned to each geographic level-statistic combination. Thus, by definition, the empirical privacy loss associated with a particular geographic level-statistic (e.g., total population count) must be less than the privacy loss budget.

*Although this assertion is true in traditional epsilon-DP, as discussed above, TopDown complicates it by introducing invariants (so, for example, the EPL of total population count at the state level is infinity). At county or enum_dist level, is EPL less than epsilon because the levels actually combine to compromise privacy?  Or is it less because the deductive proof that the algorithm is epsilon-DP relies on application of the “sequential composition theorem” which is an inequality that might include substantial slack? We have not modified the main text to attempt to elaborate on this, but we have added supplementary material with simplified examples of when EPL is very close to epsilon; when it is less, due to slack; and when it is more, due to invariants. *

- For a given value of epsilon, we can compute the portion of that value that is assigned to each geographic level - query combination. For example, epsilon of 0.25 is divided up as follows:
  Geographic levels = 0.25 to each level
  Tables = 0.1 (detailed), 0.225 (household-group quarters), 0.675 (voting age - Hispanic - race)
  We can multiply the geographic level fraction by the table fractions by epsilon to yield:
  Geog level - detailed query = 0.00625 epsilon
  Geog level - household group quarters query = 0.0140625 epsilon
  Geog level - voting age - Hispanic - race query = 0.0421875 epsilon
  These epsilons still do not equate to an epsilon associated with a particular statistic, such as total population count. Given the optimization step and the state-level total population invariant, I'm not sure if we can compute an epsilon value for a particular statistic. But these epsilon values seems like a more appropriate comparison to the empirical privacy loss reported by the authors.

 

*Thank you for this useful exposition, and we considered pivoting to these sized values in the supplementary material that explores how our EPL is constructed.  Were we to focus only on the Imprecise Histogram portion of TopDown and only consider the detailed queries at a single geographic level, we would find an EPL that precisely matches the 0.00625 epsilon you have calculated.  We believe that the value of the EPL construct is to provide an alternative to the epsilon value deduced with formal logic in cases like TopDown, where the sequential composition and invariants together might yield an empirical privacy loss that is smaller *or *larger than the proven epsilon. We eventually decided to focus on a somewhat larger epsilon in the supplementary material because we thought that this made the exposition and visuals was clearer.*

**Results - Comparison with error and privacy of simple random sampling**

- I would like to have a table of MAE and EPL values for Simple Random Sampling. Consider adding those values to the Extended Data product currently available, or adding another Extended Data product with these values.

*Good idea. We have added another supplementary table with these values.*


*  *

- Consider adding a plot of EPL by sample size to supplement or even replace the final paragraph of this subsection. There are a lot of numbers in the final paragraph, and I find it difficult to visualize the relationship between EPL and sampling fraction just by reading the numbers.

*We are hesitant to add this plot, because we want to focus the reader’s attention on the comparison of SRS EPL and TopDown EPL. Is including the supplementary table from the point above sufficient to address this request?  Then the interested reader will have all the numbers necessary to make this plot for themselves. It looks like this:*

**


*  *

- The x-axis for Figure 2 depicts values of Empirical Privacy Loss, but neither the text nor the caption describe how the values were computed. This comment fits with comment 12 - what does the Empirical Privacy Loss summary statistic mean and how was it computed.

*We have added supplementary material with a more detailed exposition of the EPL value, as well as including a short, but more precise definition in the methods section, and adding text to the caption of Figure 1, so we hope that now it is clearer what we have computed here.*

**Results - Bias in the variation introduced by TopDown**

- Figure 3 plots the error and EPL for epsilon equal to 0.5, 1.0, and 2.0, but the text in the first paragraph describes EPL by homogeneity index for epsilons of 0.25, 1.0, and 4.0. I strongly recommend making the values in the text and the plot consistent with one another. That consistency will make it easier to interpret the plot in Figure 3.

*We have changed the epsilon values described in the text to match the figure, as above.*


*  *

- I recommend moving the (Figure 3) parenthetical to the end of the discussion on EPL by homogeneity for enumeration districts. Figure 3 only shows the results for enumeration districts, but the parenthetical comes after the discussion for counties.

*Good idea, we have done this.*

- In the paragraph and Figure 3, the authors list a summary statistic for bias by homogeneity index and epsilon. Is the summary statistic the mean or the median?

*It is the arithmetic mean.  We have added to the text in the methods section and figure caption to make this clearer. *

- Figure 3 displays the violin plot/mean bias for 11 of 23 homogeneity index values. I recommend modifying the figure caption to indicate that the authors are only displaying some of the homogeneity index values on the plot.

*We have added to the caption to better explain this potentially confusing point---we have not suppressed any homogeneity index values, rather there are no enumeration districts with value zero in precisely 19 cells of the detailed histogram.*

- I also recommend modifying the x-axis label to indicate that the homogeneity index values are for enumeration districts. That would help readers immediately understand what geographic units are being plotted.

*Good idea, we have done this.*

- Is the work clearly and accurately presented and does it cite the current literature?

Yes

- Is the study design appropriate and is the work technically sound?

Yes

- Are sufficient details of methods and analysis provided to allow replication by others?

Yes

- If applicable, is the statistical analysis and its interpretation appropriate?

Partly

- Are all the source data underlying the results available to ensure full reproducibility?

Yes

- Are the conclusions drawn adequately supported by the results?

Partly

*Thank you for your work reviewing this paper!*
